# Rationale for a Rapid Methodology to Assess the Prevalence of Hearing Loss in Population-Based Surveys

**DOI:** 10.3390/ijerph16183405

**Published:** 2019-09-13

**Authors:** Tess Bright, Islay Mactaggart, Min Kim, Jennifer Yip, Hannah Kuper, Sarah Polack

**Affiliations:** 1 International Centre for Evidence in Disability, London School of Hygiene & Tropical Medicine, London WC1E 7HT, UK; islay.mactaggart@lshtm.ac.uk (I.M.); jennifer.yip@lshtm.ac.uk (J.Y.); hannah.kuper@lshtm.ac.uk (H.K.); sarah.polack@lshtm.ac.uk (S.P.); 2 Tropical Epidemiology Group, Faculty of Infectious Disease Epidemiology, London School of Hygiene & Tropical Medicine, London WC1E 7HT, UK; Min.Kim@lshtm.ac.uk

**Keywords:** hearing loss, epidemiology, developing countries

## Abstract

Data on the prevalence and causes of hearing loss is lacking from many low and middle-income countries, in part, because all-age population-based surveys of hearing loss can be expensive and time consuming. Restricting samples to older adults would reduce the sample size required, as hearing loss is more prevalent in this group. Population-based surveys of hearing loss require clinicians to be involved in the data collection team and reducing the duration of the survey may help to minimise the impact on service delivery. The objective of this paper was to identify the optimal age-group for conduct of population-based surveys of hearing loss, balancing sample size efficiencies, and expected response rates with ability to make inferences to the all-age population. Methods: Between 2013–2014, two all aged population-based surveys of hearing loss were conducted in one district each of India and Cameroon. Secondary data analysis was conducted to determine the proportion of hearing loss (moderate or greater) in people aged 30+, 40+ and 50+. Poisson regression models were developed to predict the expected prevalence of hearing loss in the whole population, based on the prevalence in people aged 30+, 40+, and 50+, which was compared to the observed prevalence. The distribution of causes in these age groups was also compared to the all-age population. Sample sizes and response rates were estimated to assess which age cut-off is most rapid. Results: Of 160 people in India and 131 in Cameroon with moderate or greater hearing loss, over 70% were older than 50 in both settings. For people aged 30+ (90.6% India; 76.3% Cameroon), 40+ (81% India; 75% Cameroon) and 50+ (73% India; 73% Cameroon) the proportions were higher. Prediction based on Poisson distributed observations the predicted prevalence based on those aged 30+, 40+, and 50+ fell within the confidence intervals of the observed prevalence. The distribution of probable causes of hearing loss in the older age groups was statistically similar to the total population. Sample size calculations and an analysis of response rates suggested that a focus on those aged 50+ would minimise costs the most by reducing the survey duration. Conclusion: Restricting the age group included in surveys of hearing loss, in particular to people aged 50+, would still allow inferences to be made to the total population, and would mean that the required sample size would be smaller, thus reducing the duration of the survey and costs.

## 1. Introduction

In 2018, the World Health Organization (WHO) estimated that 466 million people had “disabling” hearing loss globally (i.e., average of hearing thresholds at 0.5, 1, 2 and 4 kHz of ≥41 dB for adults and ≥31 dB for children) [1,2]. This figure increased from 360 million in 2012. Population growth, global ageing, and increased exposure to risks such as noise exposure, ototoxic medications, and infectious diseases are some of the likely reasons for this substantial increase [2,3]. The prevalence of hearing loss increases with age [4]. It is estimated that one third of people aged over 65 years of age are affected by moderate or greater hearing loss [5].

The impact of hearing loss on affected individuals and their family is well-established. Childhood hearing loss can adversely affect speech and language development, social and cognitive development, and school performance [3,6]. People with hearing loss are often poorer, experience lower quality of life and social isolation, and have high levels of depression and dementia [3,7]. The impact of hearing loss may be minimised if rehabilitation services, such as assistive devices, medical treatment, or sign language training, are available [8,9,10]. Access to these services is extremely limited in low and middle-income countries (LMICs), where >80% of people with hearing loss reside [1].

Reliable data on the prevalence and causes of hearing loss is lacking, particularly in LMICs, so global estimates are based on limited evidence. Stevens et al. (2013) conducted a systematic review of population-based surveys of hearing loss, identifying only 42 surveys in 29 countries worldwide [4]. Of these, only 24 were conducted in LMICs and the majority were carried out more than 10 years prior to the review. Lack of adequate data makes it difficult to plan and monitor the impact of services and advocate for increased resources. It has also likely contributed to hearing loss being overlooked in global and local health strategies [11]. As a consequence, the 2017 WHO resolution for the Prevention of Deafness and Hearing Loss urges member states to collect high-quality population-based data on the prevalence and causes of hearing loss “in order to develop evidence-based strategies and policies” to address this growing issue [12].

A key driver of the lack of data on hearing loss is the significant methodological and economic challenges that exist in carrying surveys on this topic, [4,13] and the few surveys that have been conducted have varied in methods, making comparison of results difficult. In 1999, the WHO published the Ear and Hearing Disorders survey protocol, which aimed to standardise the methodology and allow comparability between estimates and increase data collection efforts [14]. The recommended technique in the WHO protocol for assessing children <4 years is otoacoustic emissions (OAE) and auditory brainstem response (ABR) testing. For those aged ≥4 years, pure tone audiometry is recommended. To understand causes, tympanometry (test of middle ear function) and otoscopy (visual examination of the ear) are recommended for all age groups alongside questions relating to the history of hearing loss. However, these assessments can be resource intensive, requiring high-cost equipment, specialist examiners, and quiet conditions. In addition, the protocol includes survey participants of all ages, necessitating a large sample size to accurately estimate the prevalence, making the survey time-consuming and expensive. As a consequence, few surveys have used this protocol.

Alternatives to all-age population-based survey approaches to measure the prevalence of other types of impairment exist. For instance, the Rapid Assessment of Avoidable Blindness (RAAB) [15] is a rapid method that is appropriate where data are needed quickly, and time- or cost-related factors are barriers to carrying out a full epidemiological survey [16]. The RAAB is rapid and affordable for several reasons. First, it only includes people ≥50 years based on the rationale that over 80% of blindness is experienced by this age group and the causes are representative of the total population [15]. The higher prevalence in this age group reduces the sample size required. Secondly, RAAB uses a simplified eye examination protocol, reducing the time and costs of the survey. Thirdly, it uses automated data entry and analysis, eliminating the cost of specialist statistical analysis. There are additional advantages to restricting the population to ≥50 years: this population is more likely to be available during the day for data collection than younger adults, contributing to higher response rates [17]. To date, over 300 RAAB surveys have been conducted in 71 countries, and current global estimates of visual impairment are now derived from surveys using this method [18]. These types of targeted surveys are also used in the field of HIV surveillance at antenatal clinics in order to obtain an indication of population prevalence and assess trends on a regular basis and at a low cost [19].

Like visual impairment and blindness, the prevalence of hearing loss dramatically increases with age, suggesting that a comparable “rapid” protocol could be used to estimate hearing loss in the population [4]. However, this requires first assessing the proportion of hearing loss that would be captured by focusing only on an older group, rather than all-age population, as well as examining the distribution of causes in older age groups in comparison to the overall population. The purpose of such a rapid protocol would be to increase population-based data on hearing loss in a low-cost way by reducing the sample size and number of clinical tools required. It is recognised that restriction to older populations would not provide detailed data on children, which attribute a substantial amount to years lived with disability [20]. However, given the low prevalence of hearing loss among younger age groups, population-based surveys are limited in the level of detail that can be gained about children [4]. Other approaches, such as Key Informant Method studies or school screening, may be more appropriate for younger populations [21,22,23].

The aim of this paper was to consider the feasibility of a rapid population-based survey methodology for assessing the prevalence and causes of hearing loss to inform planning of ear and hearing services. Specifically, through analysis of data from previous all-age hearing surveys undertaken in India and Cameroon, we explored the data that would be captured by restricting the survey population to an older age group in terms of the proportion of hearing loss, the distribution of causes, expected response rates, and the required sample size. 

## 2. Materials and Methods

### 2.1. Survey Methodology

In 2013–2014, two all-age population-based surveys of hearing loss were conducted in India (Mahbubnagar District) and Cameroon (Fundong Health District). In the current paper, we analysed these data to estimate the prevalence and causes of hearing loss in people aged ≥30, ≥40 and ≥50 years, and compared findings to the total population. As the feasibility and cost of conducting a rapid survey also depends on the sample size and the response rate, we also computed sample size estimations and expected response rate for each of these age cut-offs.

The methods and results of these surveys have been published previously. Nevertheless, the next section provides a summary [24,25].

### 2.2. Sampling

The expected prevalence of “disabling” hearing loss (i.e., average hearing level ≥41 dB adults or ≥31 dB children) was conservatively estimated to be 4%. This required a sample of 4056, assuming a precision of 20%, 95% confidence, a design effect (DEFF; adjustment to sample size due to cluster sampling) of 1.5 and 20% non-response. A two-stage sampling procedure was used. Fifty-one clusters (e.g., villages) of 80 people each were selected using probability-proportionate-to-size sampling, using the most recent census data (2003 Cameroon; 2011 India) as the sampling frame. Census data is likely to include the vast majority of units of the population of interest (i.e., adequate coverage), provided it is fairly recent. Within clusters, households were selected using compact segment sampling. All the people in the selected households were eligible.

### 2.3. Measurement of Hearing Loss

A modified version of the WHO Ear and Hearing Disorders survey protocol was used in both surveys. Screening for hearing loss was conducted by an audiologist in a central location in each cluster. Ambient noise was measured and recorded using a sound level meter prior to testing. A quiet environment was chosen for testing with the aim of keeping the ambient noise below 40dBA as per WHO recommendations [14]. However, testing was not postponed if the ambient noise was above 40dBA for pragmatic reasons. Calibration of equipment according to standards (ISO389-1/ANSI-S3.6) was carried out prior to fieldwork.

A two-stage screening protocol was used. Initial screening of all participants was through OAE (Otocheck LE or Otoport Lite) testing. Participants aged ≥4 years who failed OAEs in both ears underwent pure tone audiometry screening (Interacoustics screening audiometer model AS608) to assess the level of hearing loss. Hearing thresholds were determined at frequencies of 1 kHz, 2 kHz, 4 kHz, 0.5 kHz in each ear. Children <4 years underwent OAE testing only.

Cases of hearing loss were defined as those with pure-tone average ≥41 dBHL in adults (age ≥ 18) and ≥31 dBHL in children (age 4–17) in the better ear, or children aged <4 who failed the OAE test in both ears. In addition, participants who could not undertake PTA, but failed OAE in both ears, were considered to have hearing loss.

### 2.4. Establishing Cause of Hearing Loss

The exact causes of hearing loss are difficult to establish even in clinical settings. This survey used a pragmatic approach to determine the “probable” causes. Cases were examined by an Ear Nose and Throat nurse (Cameroon) or an audiologist (India) who indicated the main probable cause based on otoscopy and questions about clinical history of hearing loss, derived using the original WHO survey protocol. There are three types of hearing loss: sensorineural (inner ear site of lesion; usually permanent), conductive (outer or middle ear site of lesion; usually temporary), and mixed (combination of sensorineural and conductive). We categorised the causes broadly into probable sensorineural and probable conductive hearing loss according to the following:

- Probable conductive: impacted wax, foreign body, otitis externa, chronic suppurative otitis media, otitis media with effusion, acute otitis media, and dry perforation of the tympanic membrane.
- Probable sensorineural:
  ○ Congenital: history indicative of congenital causes (infectious disease during pregnancy, genetic conditions)
  ○ Infectious conditions: history of infectious conditions that cause hearing loss (e.g., meningitis)
  ○ Noninfectious conditions: history of non-infectious conditions related to hearing loss (diabetes, noise exposure, ototoxicity)
  ○ Age-related undetermined: hearing loss present since old age (60 years +) (indicative of presbyacusis) [26].
  ○ Undetermined causes: where otoscopic examination was normal and clinical history did not provide sufficient information to classify hearing loss.

### 2.5. Rationale Study Methodology

Data from the two surveys were re-analysed using STATA (version 15.0, StataCorp LLC, College Station, TX, USA).

#### 2.5.1. Study Outcomes

Proportion of hearing loss in people aged 30+, 40+, 50+: the proportion of hearing loss (based on definitions above) in those aged 30+, 40+ and 50+ was calculated in both India and Cameroon by creating binary age group variables for each age cut-off. The prevalence of hearing loss in 10-year age bands was also calculated. To account for the clustering design, the “svy” command was used. 

Poisson regression models: In order to determine the impact of using data from older populations only to make inferences on hearing in the whole (all age) population, we created Poisson regression models based on hearing loss data from 30+, 40+ and 50+ age groups only to predict all-age population prevalence. Poisson regression is used to model response variables that are counts (integers) which follow the Poisson distribution. The shape of the distribution of prevalence by age group in the two settings follows a Poisson distribution. The predicted prevalences were then compared to the observed prevalences. If the confidence intervals overlapped, we considered the model was a good predictor. 

Causes of hearing loss in people aged 30+, 40+, 50+ compared to the total population: the causes of hearing loss in those aged 30+, 40+, 50+ compared to the total population, using the binary variables created in the first analysis. 

Sample sizes: The required sample sizes were calculated according to the prevalence of hearing loss in people aged 30+, 40+ and 50+. The purpose of this analysis was to demonstrate how the sample size changes with expected prevalence and to gain an estimate of how much the sample size reduces with different age cut-offs. The calculation was done using the expected prevalence for each age group and average DEFF for a cluster size of 40 (obtained from the datasets from each country), an expected response rate of 90%, required precision of 20% (around the estimate), and 95% confidence. The cluster size (*n* = 50) in RAAB relates to the number of people that can be feasibly examined in one day. A cluster size of 40 was chosen for the calculations because we assumed that fewer people can be feasibly examined in one day than in RAAB due to time-extensive clinical examinations. 

Response rate: Data were collected on age and sex of non-responders in both India and Cameroon. Response rates by age cut-off were calculated to assist in determining the most appropriate age group.

#### 2.5.2. Ethics Approval and Consent to Participate

All participants gave their informed consent for inclusion before they participated in the study. The study was conducted in accordance with the Declaration of Helsinki. For children under age 21 years, a caregiver was required to provide consent and to remain present throughout the screening. The protocol was approval by London School of Hygiene & Tropical Medicine Research Ethics Committee (ref: 6207), the Public Health Foundation of India Institutional Ethics Committee (ref: 84/2012), the Indian Council of Medical Research (ref: 36/ADR/2013-NCD-1), the National Ethics Committee for Research in Human Health (CNERSH, Cameroon) (ref: 2013/03/084/CNERSH/SP), and the Cameroon Baptist Convention Health Board Institutional Review Board (ref IRB2013-07). 

## 3. Results

In India, 4056 eligible people were enumerated, of whom 3573 were screened for hearing loss (response rate 87%). In Cameroon, 4104 people were enumerated, and 3567 screened for hearing loss (response rate 87%). Details of the non-responders and reasons for incomplete screening have been provided elsewhere [24,25].

### 3.1. Proportion of Hearing Loss in People Aged 30+, 40+, 50+

Table 1 provides the prevalence of hearing loss by age group and according to the different age cut points. The overall prevalence of hearing loss in India was 4.5% (95%CI = 3.8, 5.3). In total, 91% of cases were ≥30 years, 81% were ≥40 years, 73% were ≥50 years and 63% ≥60 years. The prevalence of hearing loss for persons ≥30 years was 9.3% (95%CI = 8.0, 10.8), 12.5% (95%CI = 10.6, 14.6) for those ≥40 years, and 17.4% (95%CI = 14.5, 20.7) for those ≥50 years. 

The all-age prevalence of hearing loss in Cameroon was 3.7% (95%CI = 2.8, 4.7). 76% of cases were older than 30 years, 75% were ≥40 years, 72% were ≥50 years and 66% ≥60 years. The prevalence of hearing loss for those ≥30 years was 8.9% (95%CI = 7.0, 11.3), 11.5% (95%CI = 8.9, 14.6) for those aged 40+, and 14.8% (95%CI = 11.5, 18.8) for those aged 50+.

### 3.2. Poisson Regression Models

In India, the expected prevalence was 3.8% (95%CI 3.0–4.5) based on a 50+ cut-point; 4.1% for 40+ (95%CI 3.5, 4.7); and 4.3% (95%CI 3.7, 5.0) for 30+. These estimates fall within the confidence intervals of the observed prevalence (4.5%; 95%CI 3.8–5.3). In Cameroon, the expected prevalence was 2.7% (95%CI 2.0, 3.3) based on a 50+ cut-point; 2.8 % (95%CI 2.1, 3.4) for 40+; and 2.8% (95%CI 2.2, 3.4) for 30+. These estimates are within the confidence intervals of the observed prevalence, with overlapping confidence intervals (3.7%; 95%CI 2.9, 4.8).

### 3.3. Causes of Hearing Loss in People Aged 30+, 40+, 50+ Compared to the Total Population

**Figure 1:**
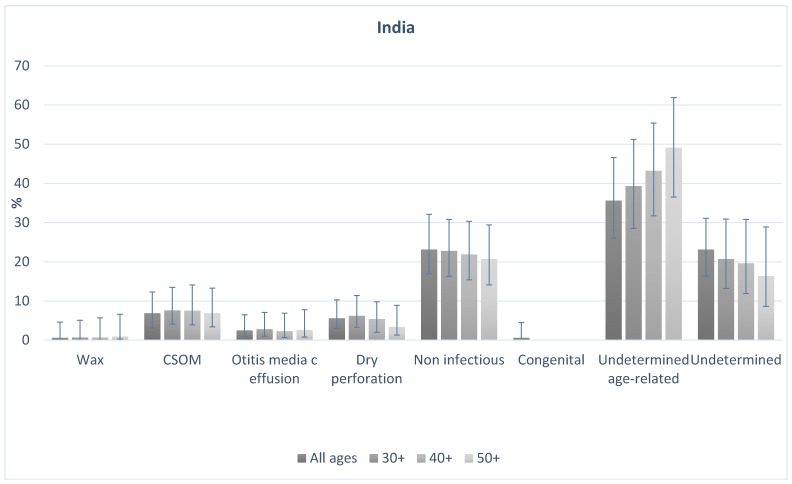
Probable causes of hearing loss in the population for all-ages and those aged 30, 40 and 50 plus in India.

**Figure 2:**
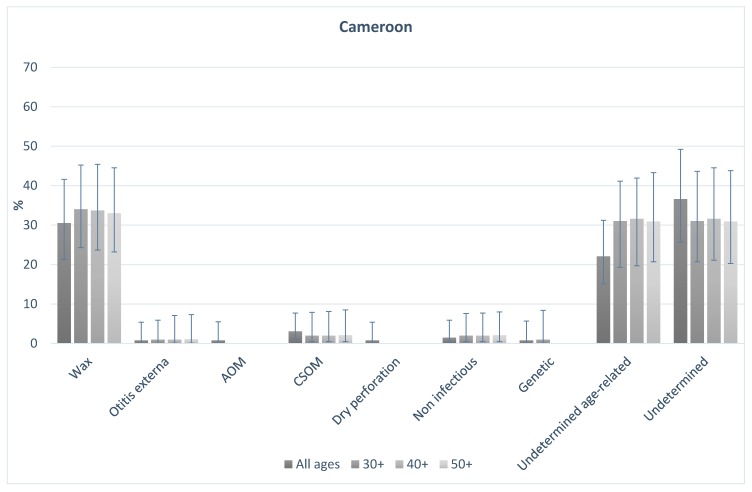
Probable CAUSES of hearing loss in the population for all-ages and those aged 30, 40 and 50 plus in Cameroon.

Figure 1 and Figure 2 shows the probable causes of hearing loss in India and Cameroon for people of all ages, and those aged 30+, 40+, and 50+. In both countries, the distribution of causes was broadly similar in older age groups in comparison to the total population. In India, 16% of all-age hearing loss was assigned to probable conductive causes. This was 17% among those aged 30+, 15% in those aged 40+, and 14% in those aged 50+. Probable sensorineural causes made up 83% of causes overall, and 83%, 85% and 86% in those aged 30+, 40+, and 50+ respectively. In Cameroon, overall 36% of causes were probable conductive, compared to 37%, 36%, and 36% in those aged 30+, 40+ and 50+ respectively. Similarly, 61% of cases were probable sensorineural overall, and 63%, 63%, 64%, in those aged 30+, 40+ and 50+ respectively. The data highlight that the proportion of causes that are likely conductive or sensorineural in nature were different in the two populations, but the distribution of the causes in the older age groups were similar to the all-age population in both settings.

### 3.4. Sample Size

Table 2 provides the required sample sizes to estimate the prevalence of hearing loss based on the different age cut-offs. All these calculations were based on a cluster size of 40, design effect of 1.5 (conservative estimate based on DEFF calculations, see Table A1), a response rate of 90%, a confidence of 95% and a precision of 20%. The required sample size decreases with increasing age cut-off; from 1560 (India) and 1597 (Cameroon) for populations aged 30+ down to 760 (India) and 907 (Cameroon) for population aged 50+. Using a cluster size of 40, the number of survey days for one survey team was calculated and is displayed in the table (sample size divided by cluster size). In India, the required sample size of 1560 for population aged 30+ equates to 39 survey days, compared to 19 survey days required for population aged 50+.

### 3.5. Response Rate

Table 3 shows that the response rate in both surveys increased with increasing age cut-off.

### 3.6. Optimal Age Cut Off for Population-Based Surveys of Hearing Loss

To assess which age-cut off was most appropriate, consideration of the following factors was made: response rate, sample size required, proportion of hearing loss missed, predictive power (based on Poisson model), causes, and expected costs. This comparison is shown in Table 4. The required sample size for a survey of people 30+ years would be approximately 1500 compared to 900 for 50+. Based on India data, the proportion of hearing loss missed if the focus was on people aged 30+ would be 10% compared to 27% for a 50+ cut-off. In Cameroon, these proportions varied less; – between 24% (30+ years) and 28% (50+years). Analysis of data from India and Cameroon showed that the response rate increased with age and was highest in those aged 50+. The Poisson models showed that the overall prevalence can be predicted using data from 30+, 40+ and 50+. Predictive power tended to increase with decreasing age cut off, as expected. Thus, there is a tradeoff between raising the age cut off and losing some predictive power. Based on the sample size and cluster size, the table shows that with a 50+ age group, the costs of the survey would be at least 77% cheaper than an all-age survey.

These findings suggest that assessment of people aged 50+ in clusters of 40 may be the most appropriate in terms of survey duration and affordability. Restricting the population would result in a substantial reduction in the sample size required to accurately estimate the prevalence, whilst still capturing the majority of hearing loss and overall cause distribution and allowing the survey to be more affordable and efficient.

## 4. Discussion

### 4.1. Summary of Results

In two population-based surveys of hearing loss in India and Cameroon, approximately 18% of the population were aged 50+, yet this age group accounted for over 70% of the prevalence of hearing loss. An age cut-off of 40+ increases this proportion to 75% and 81% in Cameroon and India respectively. People in older age groups are much more likely to have hearing loss than younger age groups, which suggests that focusing on older age groups is a valid public health approach for gathering epidemiological data on hearing loss prevalence for planning services and advocating for increased resources. Poisson regression models show that based on the 30+, 40+, and 50+ age groups, the prevalence in the total population be predicted and estimates fall within the 95% confidence interval range. The causes in the total population are comparable to the older age groups even in two settings where the proportion of causes attributed to conductive vs sensorineural hearing loss are quite different. The sample size reduces with increasing age cut-off, and the response rate increases. This may be because older people are less likely to be away from the home working when the survey team visits. Lower response rates can result in non-response bias and hence reduce the representativeness of the sample population. If lower response rates are expected, then the sample size is usually increased to account for this, increasing the resource burden for survey completion. These factors are important for reducing the time and costs of conducting population-based surveys and suggest that the 50+ age group would result in the most rapid data collection.

### 4.2. Proposal for Rapid Assessment of Hearing Loss

This paper suggests that assessment of those aged 30+, 40+ or 50+ captures the majority of people with hearing loss in population-based surveys. The advantage of restricting to these older age groups is a reduction in the sample size required to accurately estimate prevalence. Taking into consideration the proportion of hearing loss, sample size, response rate for surveys including people aged 30+, 40+, or 50+ suggests that a focus on those aged 50+ would be the most rapid, lowest cost, and have lowest demand on human resources whilst still capturing the majority of hearing loss in the population. If surveys are lower cost and have less time demand on local clinicians, they are more likely to be conducted.

A focus on adults also allows for a simplified examination, in comparison to the WHO protocol. In this age group, pure tone audiometry can be conducted without the need for more expensive tests such as ABR, and OAE testing required for children. Pure tone audiometry can now be conducted using validated smartphone-based tests which are automated [27]. The advantages of smartphone-based tests over screening audiometers in survey settings is portability, and ability for non-specialist staff to undertake the tests [28]. Therefore, the presence of audiologists in the field may not be required, although this needs further verification.

This type of survey would take a pragmatic approach to acquiring hearing loss data in a targeted and low-cost manner. Rapid surveys are not intended to replace all-age surveys, however, are useful when resource or time-constraints exist for carrying out a full survey. The survey also takes a public health approach, focusing on the population who are most likely to experience hearing loss. This data is important for informing service provision, for example the need for hearing aids.

### 4.3. Further Research Needs

Several challenges still remain in developing the methods for the rapid assessment of hearing loss, and these will require further research and testing.

#### 4.3.1. Personnel Required

Carrying out surveys can take several weeks, and participation from specialist staff for this duration could have a detrimental impact on needed service provision. Ear and hearing care professionals are scarce in most LMICs. This highlights the importance of minimising the required sample size. In addition, recent research in Malawi has shown that non-specialist health workers (e.g., nurses) can accurately assess hearing levels, however, ear examination requires prior experience to assign causes accurately (e.g., at least ENT clinical officer) [28]. Further evidence from other settings is warranted. Otoscopic examination to make a diagnosis of the causes of hearing loss often requires extensive training and experience, necessitating ENT specialist involvement. Further evidence is required to understand whether a lower cadre of health care worker can be trained to undertake otoscopy accurately to avoid the involvement of higher-level health care workers in hearing surveys. This will likely vary according to the country of research. Several previous studies have found that video-otoscopy can be used successfully to establish the presence of various pathologies from a distance [29]. Research is needed into whether this method could be feasibly used in a survey setting.

Evidence from South Africa suggests that trained community health workers can successfully carry out screening audiometry using a mobile-based test called hearScreen [30]. This result is promising, however, further evidence is required from population-based survey settings.

#### 4.3.2. Establishing Causes of Sensorineural Hearing Loss

Whilst otoscopy can help establish likely causes of conductive hearing loss, the exact causes of sensorineural or mixed hearing loss are difficult to establish even in clinical settings. This is one reason why a large proportion of causes of hearing loss are undetermined in many previous surveys [4,24,25]. There are a multitude of overlapping risk factors such as noise exposure, ototoxic drugs, ageing, and infectious diseases. Determining which factor is the main cause of hearing loss is difficult. Collecting data on causes is affected by recall bias, and potentially lack of health awareness. These factors may partly explain why the causes in all-ages vs the older age group are comparable, however, a greater level of detail on causes from surveys is currently not possible with the available screening tools. What can be obtained from surveys is a crude summary of main potential causes, which is likely to be adequate for planning purposes. To supplement this information, data could be systematically and uniformly collected on a range of associated risk factors. Algorithms may be developed to assign the most likely cause in a standardised manner. These tools need to be developed, pilot tested and adapted before use in a full survey.

Tympanometry is used to aid diagnosis of middle ear conditions such as middle ear effusion and to determine the type of hearing loss (sensorineural, or conductive). Including tympanometry in the protocol could increase the costs of a rapid survey, both in terms of the equipment, personnel and time taken to complete the test. The added value of tympanometry needs to be explored and verified, for instance, by investigating whether tympanometry allows some of the undetermined causes to be otherwise classified. In addition, further research is required to determine whether less expensive tests and portable, such as tuning forks, are adequate for this purpose.

#### 4.3.3. Exclusion of the Paediatric Population

The proposed rapid assessment method excludes the paediatric population. However, a population-based survey of hearing loss is not the ideal method for understanding childhood hearing loss. The prevalence of hearing loss in children is typically very low (<2% in India and Cameroon) which means that a large sample size (>10,000; 80% response rate; DEFF 2; 20% precision) would be required to accurately obtain prevalence data for this age group via population-based surveys. Despite the low prevalence, the impact of hearing loss on children is likely to be greater due to the impact on speech and language development, and the greater number of years lived with disability. School or newborn screening, as well as clinic-based surveys or studies using the Key Informant Method (KIM) may be more appropriate study designs for understanding hearing loss epidemiology in children [23]. RAHL does not seek to divert resources away from the paediatric population, but to provide crucially needed data on population prevalence of hearing loss in an affordable way. Other approaches such as KIM or school screening can be used to supplement RAHL data to provide paediatric specific information.

### 4.4. Limitations

The analysis presented here has some limitations that should be taken into account. The study used data from two all-age population-based surveys in distinct locations at one point in time. However, the results may not be generalisable to other settings. Further research is required to confirm the results from other settings at different time points to ensure that the cohort effect does not play a role in the findings. The models used in this paper to predict the overall prevalence in the population based on older age groups may not be applicable to other populations where age-specific prevalence is not known. This is because the distribution of the prevalence of hearing loss may not take the shape of a Poisson distribution. This warrants further attention.

## 5. Conclusions

This paper presented a rationale for a rapid assessment of hearing loss and its causes in population-based surveys. The aim of a rapid assessment of hearing loss is to estimate the prevalence and causes of hearing loss and ear disease in a resource constrained setting, in order to plan and monitor services. This paper found that assessment of people aged 30+, 40+ and 50+ provides a good indication of the prevalence and causes of hearing loss in the total population. Restricting the sample to an older age group means that the required sample size was lower, reducing the time and expense of carrying out a survey of hearing loss. The costs and duration of carrying out the survey will be particularly reduced by focusing on those aged 50+ due to the higher prevalence and expected better response rates in this group. The examination protocol may be simplified using audiometry, alongside otoscopy, and a clearly defined questionnaire. Mobile-based automated audiometry could enable non-specialist staff to carry out hearing testing, reducing the cost of the survey and the impact on usual service delivery. Further research is required to refine the method before scaling-up data collection.

## Figures and Tables

**Table 1:** Prevalence of and proportion of total hearing loss cases (≥41 dB adults; ≥31 dB children), by age group and by varying age cut points (30+; 40+; 50+) in India and Cameroon.

Age Group	India			Cameroon		
	Prevalence (%, 95%CI)	*N*	% total cases	Prevalence (%, 95%CI)	*N*	% total cases
Age group						
0–3	1.7 (0.7, 4.8)	5	3.1	1.7 (0.7, 3.5)	8	6.1
4–19	0.3 (0.1, 0.9)	4	2.5	1.1 (0.7, 1.9)	18	13.7
20–29	0.9 (0.3, 2.9)	6	3.8	1.3 (0.5, 3.6)	5	6.1
30–39	2.6 (1.4, 4.9)	13	8.1	0.7 (0.1, 3.0)	2	1.5
40–49	4.1 (2.5, 6.6)	16	10.0	1.8 (0.5, 5.9)	4	3.0
50–59	5.1 (3.1, 8.1)	16	10.0	3.4 (1.3, 8.4)	7	5.3
60–69	21.8 (16.8, 27.8)	51	31.9	8.0 (4.8, 13.1)	15	12.2
70+	41.5 (32.6, 51.0)	49	30.6	29.8 (23.4, 37.0)	72	57.2
Total	4.5 (3.8, 5.3)	160	100	3.7 (2.8, 4.7)	131	100
Varying age cut points						
Total 30+	9.3 (8.0, 10.8)	145	90.6	8.9 (7.0, 11.3)	100	76.3
Total 40+	12.5 (10.6, 14.6)	130	81.3	11.5 (8.9, 14.6)	98	74.8
Total 50+	17.4 (14.5, 20.7)	116	72.5	14.8 (11.5, 18.8)	94	71.8
Total 60+	28.4 (24.5, 32.7)	100	62.5	20.3 (16.3, 25.0)	87	66.4

CI = confidence interval.

**Table 2:** Estimated sample size for varying age cut-offs in India and Cameroon and the estimated number of survey days (Using average DEFF of 1.5, cluster size of 40).

Age	Prevalence (%) India	Sample Size	No. of Days	Prevalence (%) Cameroon	Sample Size	NO. OF DAYS
All ages	4.5	3390	85	3.7	4643	116
30+	9.3	1560	39	9.1	1597	40
40+	12.5	1120	28	11.7	1207	30
50+	17.4	760	19	15.0	907	23

**Table 3:** Response and non-response by age group.

Age	India				Cameroon			
	Response		Non-Response		Response		Non-Response	
**Age group (years)**	N	%	N	%	N	%	N	%
**All ages**	3688	86.5	574	13.5	3673	87.1	545	12.9
**30+**	3412	92.7	269	7.3	3307	93.6	226	6.4
**40+**	3300	94.9	178	5.1	3423	95.3	159	4.7
**50+**	3189	96.7	108	3.3	3174	98.9	103	3.1

**Table 4:** Comparison of survey attributes for different age cut offs: 30+; 40+; 50+ years.

Attribute	Age Group			
	All-age	30+	40+	50+
Response rate	87%	93–94%	95%	97–99%
Approximate sample size *	4000	1500	1200	900
Proportion of hearing loss missed	0%	10–24%	18–25%	27–28%
Predicted all-age prevalence	-	Within confidence limits of observed prevalence	Within confidence limits of observed prevalence	Within confidence limits of observed prevalence
Causes	-	Representative of the total population	Representative of total population	Representative of total population
Costs **	≥100 survey days	≥40 survey days ≥60% cheaper than all-age	≥30 survey days ≥70% cheaper than all-age	≥23 survey days ≥77% cheaper than all-age

* Based on calculations from Table 4 (95% confidence, 20% precision, 90% response rate, varying prevalence and DEFF). ** Based on sample size calculations in Table 4, and cluster size of 40 (will vary depending on expected prevalence).

## Appendix A

**Table A1:** Probable causes of hearing loss in the population for all-ages and those aged 40 and 50 plus.

Cause	India								Cameroon							
	All ages (*N* = 160)		Age 30+ (*N* = 145)		Aged 40+ (*N* = 132)		Aged 50+ (*N* = 116)		All ages (*N* = 131)		Age 30+ (*N* = 100)		Aged 40+ (*N* = 98)		Aged 50+ (*N* = 94)	
	*N*	% (95%CI)	*N*	% (95%CI)	*N*	% (95%CI)	*N*	% (95%CI)	*N*	% (95%CI)	*N*	% (95%CI)	*N*	% (95%CI)	*N*	% (95%CI)
Probable conductive causes																
Impacted wax	1	0.6 (0.08,4.6)	1	0.7 (0.08,5.09)	1	0.7 (0.09, 5.7)	1	0.9 (0.1, 6.4)	40	30.5 (21.3, 41.6.)	34	34.0 (24.3, 45.2)	33	33.7 (23.7, 45.4)	31	33.0 (23.2, 44.5)
Otitis Externa	0	-	0	-	0	-	0	-	1	0.8 (0.1, 5.4)	1	1.0 (0.1, 6.9)	1	1.0 (0.1, 7.1)	1	1.1 (0.1, 7.3)
AOM	0	-	0	-	0	-	0	-	1	0.8 (0.1, 5.5)	0	-	0	-	0	-
CSOM	11	6.9 (3.1, 12.3)	11	7.6 (4.1, 13.5)	10	7.5 (3.9, 14.1)	8	6.9 (3.4, 13.3)	4	3.1 (1.2, 7.7)	2	2.0 (0.5, 7.9)	2	2.0 (0.5, 8.1)	2	2.1 (0.5, 8.5)
OME	4	2.5 (0.9,6.5)	4	2.8 (1.0, 7.1)	3	2.3 (0.7, 6.9)	3	2.6 (0.8, 7.8)	0	-	0	-	0	-	0	-
Dry perforation	9	5.6 (3.0, 10.3)	9	6.2 (3.3, 11.4)	6	5.4 (2.0, 9.8)	4	3.4 (1.3, 8.9)	1	0.8 (0.1, 5.4)	0	-	0	-	0	-
Total	25	15.6 (10.7, 22.3)	25	17.2 (11.8, 24.5)	20	15.2 (10.1, 22.1)	16	13.8 (8.8, 21.1)	47	35.9 (27.1)	37	37.0 (28.2, 46.8)	35	35.7 (27.6, 47.0)	34	36.2 (27.0, 46.4)
Probable sensorineural causes																
Non-infectious	37	23.1 (17.0, 32.1)	33	22.8 (16.3, 30.8)	29	21.9 (15.4, 30.3)	24	20.7 (14.1, 29.4)	2	1.5 (0.4, 5.9)	2	2.0 (0.5, 7.6)	2	2. (0.5, 7.7)	2	2.1 (0.5, 8.0)
Infectious	0	-	0	-	0	-	0	-	1	0.8 (0.09, 5.8)	0	-	0	-	0	-
Genetic	0	-	0	-	0	-	0	-	1	0.8 (0.09, 5.7)	1	1.0 (0.1, 7.4)	0	-	0	-
Congenital	1	0.6 (0.08, 4.5)	0	-	0	-	0	-	0	-	0	-	0	-	0	-
Undetermined age-related *	57	35.6 (26.0, 46.6)	57	39.3 (28.5, 51.2)	57	43.2 (31.7, 55.4)	57	49.1 (36.5, 61.9)	29	22.1 (15.1, 31.2)	29	29.0 (19.3, 41.1)	29	29.6 (19.7, 41.9)	29	30.9 (20.7, 43.3)
Undetermined	37	23.1 (16.3, 31.8)	30	20.7 (13.2, 30.9)	26	19.6 (11.9, 30.8)	19	16.4 (8.6, 28.9)	48	36.6 (25.7, 49.2)	31	31.0 (20.7, 43.6)	31	31.6 (21.1, 44.5)	29	30.9 (20.3, 43.8)
Total	132	82.5 (75.9, 87.6)	120	82.8 (75.5, 88.2)	112	84.8 (77.9, 89.9)	100	86.2 (78.9, 91.2)	81	61.0 (50.6, 71.8)	63	63.0 (53.2, 71.8)	62	63.3 (53.0, 72.4)	60	63.8 (53.6, 73.0)
Missing data	3	1.8	0	-	0	-	0	-	3	2.3	0	-	0	-	-	-
